# ECMO support may be associated with improved survival in tuberculosis associated severe ARDS

**DOI:** 10.1186/s12890-024-03356-4

**Published:** 2024-10-24

**Authors:** Bahar Nalbant, Alix Buhlmann, Lennart Wild, Christian Bode, Sascha David, Benjamin Seeliger, Klaus Stahl, for the BonHanZA study group, Bahar Nalbant, Alix Buhlmann, Lennart Wild, Christian Bode, Sascha David, Benjamin Seeliger, Klaus Stahl, Thorben Pape, Jannik Ruwisch, Marius M. Hoeper, Pedro David Wendel-Garcia, Daniel A. Hofmaenner, Rolf Erlebach, Mattia Mueller, Rea Andermatt, Konrad Peukert, Andrea Sauer

**Affiliations:** 1 Department of Respiratory Medicine, Hannover Medical School, Hannover, Germany; 2 Biomedical Research in End-Stage and Obstructive Lung Disease (BREATH), Hannover Medical School (MHH), German Center for Lung Research (DZL), Hannover, Germany; 3 Institute of Intensive Care Medicine, University Hospital Zurich, Raemisstraße 100, Zurich, 8091 Switzerland; 4 Department of Anaesthesiology and Intensive Care Medicine, University Hospital Bonn, Bonn, Germany; 5 Department of Nephrology and Hypertension, Hannover Medical School, Hannover, Germany; 6 Department of Gastroenterology, Hepatology, Infectious Diseases and Endocrinology, Hannover Medical School, Carl-Neuberg-Str.1, Hannover, 30625 Germany

**Keywords:** Respiratory failure, Membrane Oxygenation, Tuberculosis

## Abstract

**Background:**

Data describing outcome of extracorporeal membrane oxygenation (ECMO) support in Tuberculosis (Tbc)-associated acute respiratory distress syndrome (ARDS) remain sparce and are mostly confined to singular case reports. The aim of this case series was to analyze intensive care unit (ICU) survival in patients with Tbc-associated ARDS receiving veno-venous (vv-) ECMO support and to compare those to patients not receiving ECMO.

**Case presentation:**

ICU survival was analyzed retrospectively in 14 patients treated for Tbc-associated ARDS at three ECMO-referral university hospitals (Hannover Medical School, University Hospital Bonn (both Germany) and University Hospital Zurich (Switzerland)) during the last 14 years, of which eight patients received additional vv-ECMO support and six standard care only.

ICU survival was significantly higher in patients receiving additional vv-ECMO support (62.5%, *n* = 5/8) compared to those that did not (16.7%, *n* = 1/6) (*p* = 0.021). ECMO support was associated with reduced ICU mortality (Hazard ratio adjusted for baseline SOFA score [adj. HR] 0.125 (95% confidence interval (CI): 0.023–0.689), *p* = 0.017). Median (IQR) time on ECMO and invasive ventilation in the vv-ECMO group were 20 (11–26) and 37 (27–53) days, respectively. Major bleeding defined as transfusion requirement of 4 units of blood or more or surgical and/or radiologic intervention occurred only in one patient, in whom pulmonary bleeding was fatal. Thromboembolic events occurred in none of the vv-ECMO patients.

**Discussion and conclusions:**

This retrospective analysis from three large ECMO centers with similar SOPs suggests vv-ECMO support as a feasible approach in patients with severe Tbc-associated ARDS. Although affiliated with extended runtimes, vv-ECMO might be associated with improved survival in those patients. Vv-ECMO support should thus be considered in Tbc-associated ARDS to enable lung protective strategies during prolonged lung recovery.

## Background

Recently Idris et al. described in *the Journal* outcome of patients with tuberculosis (Tbc) associated critical illness, including those with severe acute respiratory distress syndrome (ARDS), supported by extracorporeal membrane oxygenation (ECMO) [1]. In this systematic review and metanalysis of 43 patients receiving diverse modes of ECMO support, the authors reported encouraging clinical outcomes with an overall intensive care unit (ICU) survival of 81.4%. Data describing outcome of ECMO support in Tbc-associated ARDS in general remain sparce and are mostly confined to only singular case reports [2]. However, the analysis by Idris et al. has some important limitations. First, patients were included based on singular case reports extracted over a wide time-period of 47 years, given rise to both reporting bias and significant heterogeneity in selection and provision of treatment as well as technology. Second, both patients with primarily respiratory as well as circulatory failure, consequentially receiving diverse modes of ECMO cannulation (vv-, va- and vav-ECMO), were included and analyzed together, thus further increasing clinical heterogeneity despite completely different primary clinical disorders.

## Case presentation

Our study-group has therefore analyzed ICU survival in a more homogenous cohort of patients treated for Tbc-associated ARDS at three ECMO-referral university hospitals (Hannover Medical School, University Hospital Bonn (both Germany) and University Hospital Zurich (Switzerland)) during the last 14 years. A total of 14 patients with Tbc-associated ARDS were included in this present case series, of whom eight received additional veno-venous (vv)-ECMO support and six received standard care only. All patients had a definitive Tbc diagnosis by positive culture of M. tuberculosis from respiratory material (13/14 bronchoalveolar lavage, 1/14 sputum). In 10/14 (71.4%) of patients PCR testing was also positive. Typical radiographic signs of pulmonary Tbc were present in 9/14 (64.3%) of patients with only unspecific radiographic findings of ARDS being present in the minority of 5/14 (35.7%) of patients. The majority of patients (11/14, 78,6%) received a classical quadruple anti-Tbc therapy consisting of Rifampicin, Ethambutol, Isoniazid und Pyrazinamide. One patient received a therapy consisting of Badequilin, Linezolid, Pretomanid and Clofazimin. One patient received a therapy consisting of Isoniazid, Pyrazinamide, Ethambutol, Rifabutin, Moxifloxacin. Another patient received no anti-Tbc therapy due to severe liver dysfunction at presentation.

Baseline demographic and clinical characteristics at implementation of invasive ventilation were comparable between the two groups (Table 1). Three patients had HIV infection, two patients received medical immunosuppression, one had a congenital immune defect and five patients had chronic alcohol abuse. Patients later receiving vv-ECMO support had significantly lower oxygenation index (Median (Interquartile range (IQR)) 71 (54–92) vs. 188 (146–236) mmHg, *p* < 0.001). Median (IQR) time to ECMO implantation was 2 (1–4) days.

**Table 1:** Demographic and clinical characteristics at start of invasive ventilation

**Category**	**Median** (interquartile range) / **No** (%)		
	**ECMO** (*n* = 8)	**no ECMO** (*n* = 6)	***P***
Age—y	39 (27–48)	44 (35–68)	0.218
Sex—no (%)			0.733
Male	6 (75)	4 (66.7)	
Female	2 (25)	2 (33.3)	
BMI—kg/m^2^	19.9 (17.6–24.6)	22.2 (20–25.4)	0.61
Immunosuppression—no (%)	3 (37.5)	4 (66.7)	0.28
MDR-Tbc—no (%)	1 (12.5)	1 (16.7)	0.825
pO_2_/FiO_2_—mmHg	71 (54–92)	188 (146–236)	< 0.001
pCO_2_—mmHg	49 (44–70)	56 (40–61)	0.707
Vasopressor therapy—no (%)	7 (87.5)	5 (83.3)	0.825
Vasopressor dose—ug/kg/min	0.38 (0.2–1.0)	0.33 (0.245–1.455)	0.613
Renal replacement therapy—no (%)	1 (12.5)	2 (33.3)	0.347
SOFA score—points	9 (7–10)	9 (8–11)	0.799
Lactate—mmol/l	1.4 (1.3–4.5)	4 (2.1–6.7)	0.228

Categorical variables are represented by number (n) and percentage (%), while continuous variables are expressed as median (25% to 75% Interquartile Range [IQR]). The normal distribution was checked using the D’Agostino-Pearson omnibus normality test and the Shapiro–Wilk normality test. Two-tailed *p* values of less than 0.05 were considered to indicate statistical significance. Comparisons of population characteristics between the ECMO and the no-ECMO group were performed using unpaired t-tests, Mann–Whitney tests and χ^2^ tests, as appropriate. All reported *p*-values are two-sided unless indicated otherwise; *p*-values < 0.05 were considered statistically significant

*ARDS* Acute respiratory distress syndrome, *ECMO* Extracorporeal membrane oxygenation, *MDR* Multi drug resistant, *SOFA* Sequential Organ Failure Assessment score, *Tbc* Tuberculosis

**Fig. 1:**
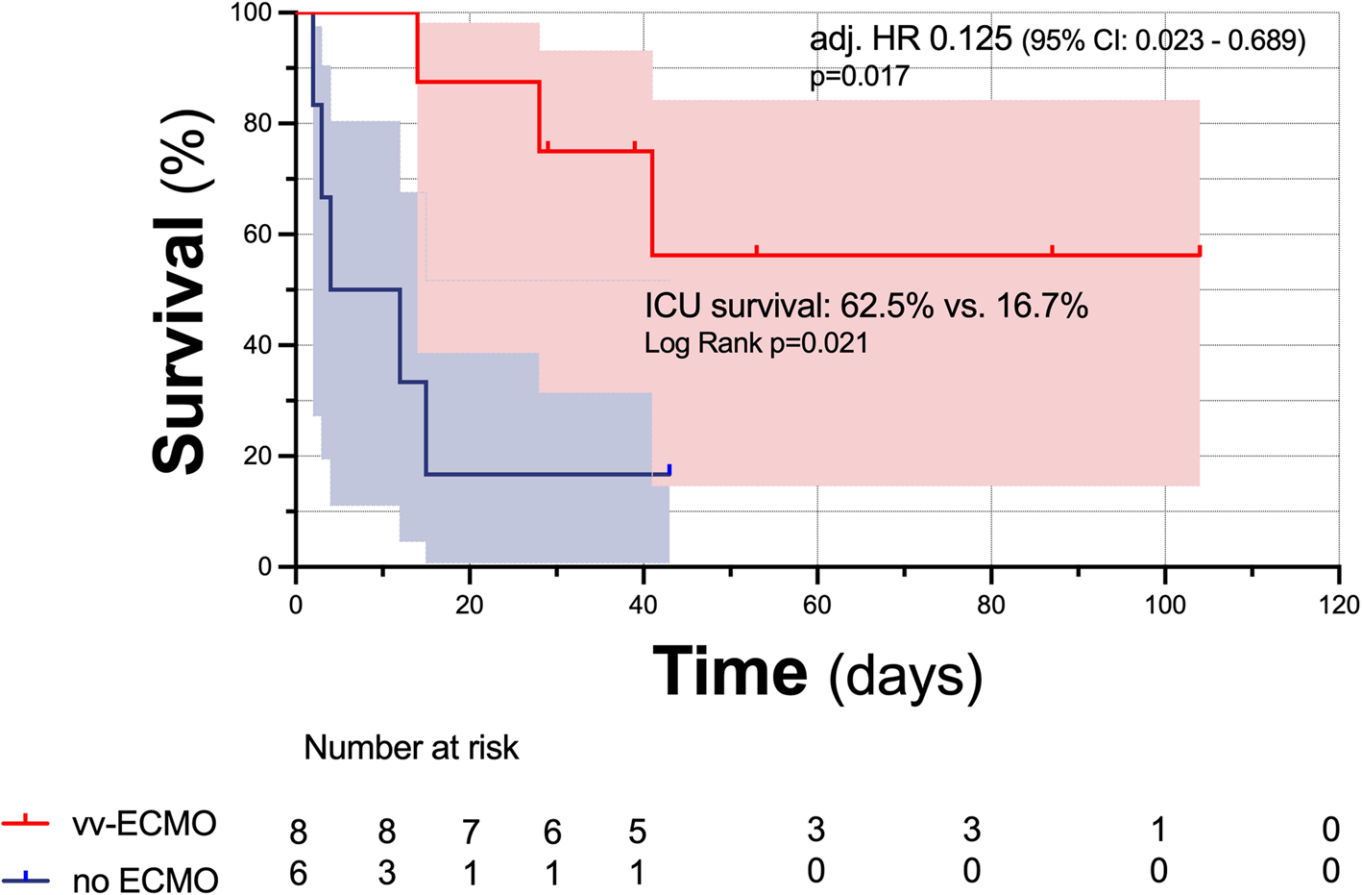
Intensive care unit survival in patients with and without vv-ECMO support for Tbc-associated ARDS. ICU survival following vv-ECMO decannulation was analyzed for the whole cohort and for the subgroups vv-ECMO vs. no-ECMO, respectively. Survival was visualized using Kaplan–Meier plots and analysed using the log-rank test. Influence of vv-ECMO support on ICU survival- was analyzed by means of uni- and multivariate Cox-proportional hazard regression models. All reported *p*-values are two-sided unless indicated otherwise; *p*-values < 0.05 were considered statistically significant. GraphPad Prism (Version 10.0, GraphPad Software, La Jolla, CA) and IBM SPSS Statistics (Version 25.0, IBM Corp., Armonk, NY) were used for data analysis and graph generation. ARDS – Acute respiratory distress syndrome, ECMO – Extracorporeal membrane oxygenation, CI – Confidence Interval, HR – Hazard ratio

ICU survival was significantly higher in patients receiving additional vv-ECMO support (62.5%, *n* = 5/8) compared to those that did not (16.7%, *n* = 1/6) (Fig. 1, *p* = 0.021). Vv-ECMO support was associated with reduced ICU mortality (Hazard ratio adjusted for baseline SOFA score [adj. HR] 0.125 (95% confidence interval (CI): 0.023–0.689), *p* = 0.017). Median (IQR) time on vv-ECMO and invasive ventilation in the ECMO group were 20 (11–26) and 37 (27–53) days, respectively. Major bleeding defined as transfusion requirement of 4 units of blood or more or surgical and/or radiologic intervention occurred only in one patient, in whom pulmonary bleeding was fatal. Thromboembolic events occurred in none of the vv-ECMO patients. In all of the six surviving patients Tbc became culture negative at a median (IQR) of 56 (29–247) days. None of the patients had a relapse during the further follow up time so far.

## Discussion and conclusions

In the meta-analysis by Idris et al. 30 patients had ARDS, 27 were supported by vv-ECMO, of which 23 (85.1%) survived. However, outcomes were reported from a multitude of different centers dating back to 1975, making a reporting bias likely. Our outcome results with a survival of about 60% generated from three centers in a recent time period and similar standard operating procedures in the ECMO cohort are better in line with the more recent ECMO trials such as the CESAR [3] and EOLIA [4] study with regards to mortality. Idris et al. did not report on patients with Tbc-associated ARDS, treated at the same institutions but without ECMO support. The finding of an exceedingly high mortality in patients without vv-ECMO support from this present cohort is provocative, but certainly needs confirmation in prospective studies. Of note, both vv-ECMO runtime and ventilator-days were excessively long in either Idris et al. and our group, underlining the slow recovery from Tbc-associated ARDS [5] and the consequential need to optimize lung-protective strategies in this cohort.

This study has substantial limitations, mainly its retrospective design and the small sample size. Survival was compared to only six patients in the SOC group. However, no patients with Tbc-ARDS have been excluded from this analysis, further highlighting the rarity of this ARDS subpopulation. Since the decision which patient received additive vv-ECMO support was not made in a randomized fashion but rather on a case-by-case basis by the treating physician team, this study certainly has the risk of a selection bias. Although utilization of additional vv-ECMO support as well as fatal outcomes were about evenly distributed over the study period, advances in the treatment of patients with severe ARDS over the last years may introduce a further bias to the generalizability of the study results.

In summary, this retrospective analysis from three large ECMO centers with similar SOPs suggests vv-ECMO support as a feasible approach in patients with severe Tbc-associated ARDS. Although affiliated with extended runtimes, vv-ECMO might be associated with improved survival in those patients. Vv-ECMO support should thus be considered in Tbc-associated ARDS to enable lung protective strategies during prolonged lung recovery.

## Data Availability

The datasets used and analyzed are during the current study are available from the corresponding author on reasonable request.

## Abbreviations

ARDS: Acute respiratory distress syndrome
CI: Confidence Interval
ECMO: Extracorporeal membrane oxygenation
HR: Hazard Ratio
ICU: Intensive care unit
MDR: Multi drug resistant
SOFA: Sequential Organ Failure Assessment score
SOP: Standard operating procedure
Tbc: Tuberculosis
Va: Veno-arterial
Vav: Veno-arterial-venous
Vv: Veno-venous
